# Circular RNA hsa_circ_0007364 increases cervical cancer progression through activating methionine adenosyltransferase II alpha (MAT2A) expression by restraining microRNA-101-5p

**DOI:** 10.1080/21655979.2020.1832343

**Published:** 2020-11-02

**Authors:** Hongfei Chen, Bin Gu, Xiang Zhao, Yupeng Zhao, Shuning Huo, Xiang Liu, Huihong Lu

**Affiliations:** Department of Anesthesia, Shanghai East Hospital, Tongji University School of Medicine, Shanghai, China

**Keywords:** Cervical cancer, hsa_circ_0007364, miR-101-5p, MAT2A

## Abstract

Emerging evidence suggested that circular RNAs (circRNAs) play critical roles in cervical cancer (CC) progression. However, the roles and molecular mechanisms of hsa_circ_0007364 in the tumorigenesis of CC remain unclear. In the present study, we used bioinformatics analysis and a series of experimental analysis to characterize a novel circRNA, hsa_circ_0007364 was up-regulated and associated with advanced clinical features in CC patients. Hsa_circ_0007364 inhibition notably suppressed the proliferation and invasion abilities of CC cells in vitro and reduced tumor growth in vivo. Moreover, hsa_circ_0007364 was uncovered to sponge miR-101-5p. Additionally, methionine adenosyltransferase II alpha (MAT2A) was verified as a target gene of miR-101-5p, and its downregulation reversed the inhibitory effects of hsa_circ_0007364 knockdown on CC progression. Therefore, we suggested that hsa_circ_0007364 might serve as an oncogenic circRNA in CC progression by regulating the miR-101-5p/MAT2A axis, which provides a potential therapeutic target to the treatment.

Research highlights

1. hsa_circ_0007364 was upregulated in CC
2. hsa_circ_0007364 promoted CC cell progression
3. hsa_circ_0007364/miR-101-5p/MAT2A axis in CC

## Graphical abstract

**Figure:**
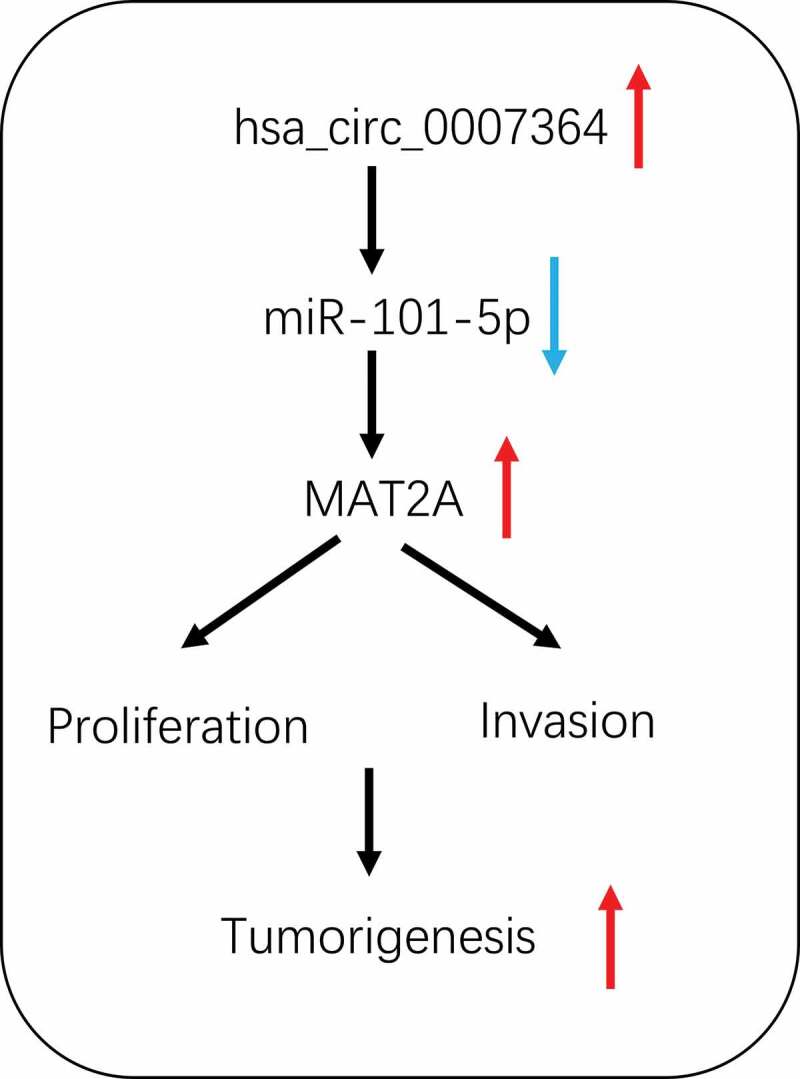

## 1. Introduction

Cervical cancer (CC) is one of the commonest gynecological malignancies. In 2012 there were an estimated 530,000 CC cases and about 275,000 CC deaths [1,2]. Despite recent advancements in cancer diagnosis and treatment, advanced CC is still associated with worse survival [3,4]. Thus, novel biomarkers and therapeutic targets are needed to improve CC outcomes.

Circular RNA (circRNA) has attracted immense attention recently, particularly regarding their role in cancer development [5,6]. This RNA species possesses a loop structure that is covalently joined and lacking the 5ʹ cap and 3ʹ polyadenylated tail, which makes them more stable than linear RNAs [7]. Increasing evidences show that circRNAs influence CC progression. Jiao et al. [8] found that hsa_circ_0000745 upregulation in CC promotes the multiplication and metastasis of cells *in vitro*. Hu et al. [9] have reported that circ_0067934 overexpression promotes the progression of CC by regulating the miR-545/EIF3C axis. According to Qian et al. [10], circRNA HIPK3 promotes CC EMT by sponging miR-338-3p, thereby upregulate HIF-1α.

MiR-101-5p is a miRNA shown to play an essential role in multiple cancers. For example, Yamada et al. [11] showed that miR‐101‐5p acted as an antitumor miRNA in clear cell renal cell carcinoma cells. Chen et al. [12] revealed that miRNA-101-5p suppressed lung cancer cell growth and aggressiveness via targeting CXCL6. Furthermore, Shen et al. [13] found that miRNA-101-5p reduced CC cells’ proliferation and invasion through suppressing CXCL6. However, the underlying molecular mechanisms of miRNA-101-5p in CC progression remain unclear.

Methionine adenosyltransferase 2A (MAT2A) is a key regulator in cellular metabolism and catalyzes the reaction of L-methionine and adenosine triphosphate (ATP) to S-adenosylmethionine (SAM) [14]. Recently, increasing studies showed that MAT2A play important roles in cancer progression. For example, Wang et al. [15] showed that reduced MAT2A expression was able to inhibit hepatocelluar carcinoma cell growth in vitro. Lo et al. [16] showed that miR-21-3p suppressed HepG2 cell growth and induces apoptosis by targeting MAT2A. However, the roles and underlying mechanism of MAT2A in CC are still unclear.

Here, we analyzed circRNAs microarray data from CC cell lines and tissues, which revealed that hsa_circ_0007364 is significantly overexpressed. Also, hsa_circ_0007364 upregulation was correlated with advanced CC features and promoted CC cell proliferation and invasion. Mechanistically, our data indicated that hsa_circ_0007364 may sponge miR-101-5p, thereby upregulating MAT2A expression and consequently promoting CC progression. Therefore, these data suggested that hsa_circ_0007364 could act as a potential therapeutic target for CC treatment.

## 2. Materials and methods

### 2.1. CC samples

Fifty-three paired CC tissues and adjacent non-tumor tissues were extracted from patients during surgery, which was conducted at Shanghai East Hospital from 2016 to 2018. The CC patients had not received any treatment prior to surgery. The clinicopathological diagnosis was determined by two pathologists according to the guidelines of International Federation of Gynecology and Obstetrics (FIGO). Ethical approval for this study was given by Research Review Board of Shanghai East Hospital. Written informed consent was received from all participants. Clinical features of CC patients are shown in Table 1.

**Table 1.** The clinical features of cervical cancer patients

Characteristics		Number
Age	<50	19
	≥50	34
Tumor size (cm)	<4	28
	≥4	25
FIGO stage	I/II	21
	III/IV	32
Lymph-node metastasis	No	29
	Yes	24
Depth of invasion	<1/2	30
	≥1/2	23

### 2.2. Cell culture and transfection

The normal human cervical epithelial cell line (End1/E6E7), and CC cell lines (HeLa, CaSki, SiHa, C-33A, C-4I, SW756), were procured from the American Type Culture Collection (ATCC). The cells were grown in DMEM (Invitrogen, Carlsbad, CA, USA) containing 10% FBS (Hyclone, Logan, UT, USA) at 37°C, 5% CO_2_, in a humified incubator.

siRNA against hsa_circ_0007364 (si-circ1#: 5′-ATCCACGTTCTAGTTTTTCGT-3′; si-circ2#: 5′-GTTCTAGTTTTTCGTTGGAAT-3′; si-circ1#: 5′-AGGAATCCACGTTCTAGTTTT-3′;) and control (si-NC), MAT2A overexpression plasmid, and control were made by GenePharma (Shanghai, China). miR-101-5p mimics or inhibitors and controls were purchased from Ribobio (Guangzhou, China). Lipofectamine 3000 (Invitrogen) was used for cell transfection.

### 2.3. Cell proliferation assays

The proliferative ability of cells was examined using cell counting kit-8 (CCK-8) and colony formation assays. For CCK-8 assay, 5x10^4^/well transfected CC cells were seeded onto a 96-well plate, then cultured for 24, 48, 72, and 96 hours. 10 μL of CCK-8 solution was then added into each well. After 2 hours, absorbance (450 nm) was read on a microplate reader (Bio-Teck). Colony formation assays were done as previously described [17].

### 2.4. Transwell assay

Cell invasion assays were done on transwell chambers (Costar, Cambridge, MA, USA). Transfected CC cells in serum-free medium were seeded onto the upper chamber coated with matrigel (BD Biosciences). DMEM containing 10% FBS was added in the lower chamber. The cells were then grown for 24 hours after which cells invading the lower chamber were fixed with 5% glutaraldehyde and stained with crystal violet (Sigma) then counted under an inverted microscope.

### 2.5. *Quantitative real-time PCR* RT-qPCR

RNA extraction was done using Trizol (Invitrogen) with RNeasy Mini Kit (Qiagen, Shanghai, China). After RNase R treatment, transcription was done using Prime Script RT Master Mix (Thermo Fisher Scientific). RT-qPCR was done on 7500 Real-Time PCR System (Applied Biosystems) using SYBR Select Master Mix (Applied Biosystems). Hsa_circ_0007364 and MAT2A levels were normalized to GAPDH expression using 2^−ΔΔCt^ method. miR-101-5p levels were normalized to U6.

Hsa_circ_0007364 circular structure was confirmed by RNase R (Geneseed, Guangzhou, Guangzhou, China) treatment, which can digest linear RNA molecules, but not circular ones [18]. Total RNA was incubated with RNase R in a 10 μL volume. After RNase R inactivation, treated RNAs were used for RT-qPCR.

### 2.6. Luciferase reporter assay

Hsa_circ_0007364-WT (wildtype), hsa_circ_0007364-Mut (mutant hsa_circ_0007364), MAT2A-WT (MAT2A bearing the wildtype putative miR-101-5p binding site) and MAT2A-Mut (MAT2A bearing mutant putative miR-101-5p binding site) were inserted into the pGL3 basic vector (Promega, Madison, WI, USA). They were then co-transfected with miR-101-5p mimics or miR-NC mimics. Forty-eight hours post-transfection, luciferase activity was assessed as per the methods described by the manufacturer and previous study [19].

### 2.7. RNA immunoprecipitation (RIP)

RIP assays were performed to probe the binding between hsa_circ_0007364 and miR-101-5p. This analysis used the EZ-Magna RIP RNA-binding protein immunoprecipitation kit (Millipore, Burlington, MA, USA) and previous studies [20,21].

### 2.8. Tumor xenograft in vivo

BALB/c athymic mice (female, 5-week-old, ~20 g) were purchased from Charles River Laboratories (Beijing, China), and housed in specific pathogen-free microisolator cages. 3 × 10^6^ cells were transfected with sh-circRNA or control were injected into mice (n = 4/group) via subcutaneous inoculation. Tumor volume was monitored every week and analyzed according to the formula volume = 0.5 × length × width2. After 6 weeks, mice were euthanized using 5% isoflurane. Tumor tissues were collected and weighed. The animal experiments were in line with guidelines of the National Institutes of Health guide for the Care and Use of Laboratory animals, and approved via the Institutional Animal Care and Use Committee of Shanghai East Hospital.

### 2.9. Actinomycin D and RNase R treatment

CC cells were stimulated with 2 mg/mL Actinomycin D (Solarbio, Beijing, China), and then the expression of hsa_circ_000736 and PTP4A2 mRNA was detected by qRT-PCR at specified times. In addition, 2 μg of RNA was incubated with or without RNase R (Solarbio) for 30 min, and hsa_circ_000736 and PTP4A2 mRNA levels were measured using qRT-PCR.

### 2.10. Statistical analysis

All the analyses were performed using SPSS 21.0 (IBM). Statistical significance between two groups was analyzed using student’s t-test, whereas one-way analysis of variance (ANOVA) with Tukey’s post hoc test was used for multiple groups. Correlations were analyzed by Pearson’s correlation coefficient analysis. The survival rate was calculated by the Kaplan-Meier method with log-rank test. Statistical significance was set at *p* < 0.05.

## 3. Results

### 3.1. Hsa_circ_0007364 is overexpressed in CC

**Figure 1.**
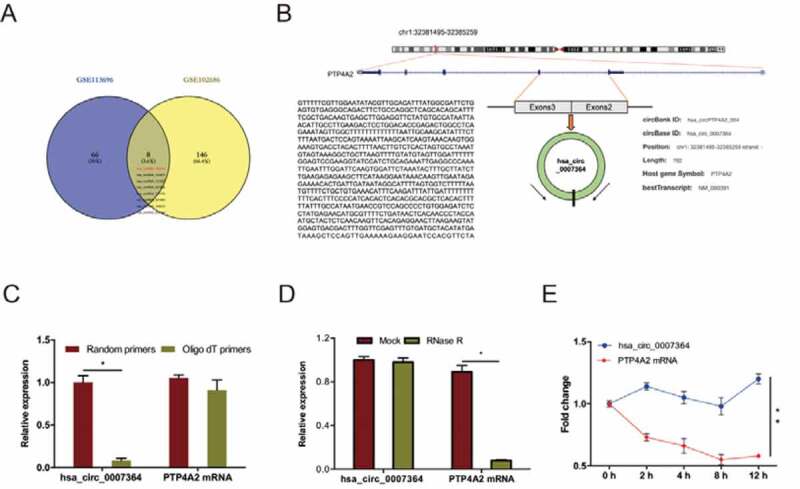
Identification of hsa_circ_0007364 as a novel cirRNA in CC. (a) The results of Venn diagram. Overlapping sections show circRNAs that are upregulated in the 2 microarray datasets. (b) Schematic illustration of hsa_circ_0007364 formation. (c) RT-qPCR analysis hsa_circ_0007364 (circPTP4A) and PTP4A2 mRNA using template cDNA reverse-transcribed using oligo dT and random primers. (d, e) RT-qPCR analysis of hsa_circ_0007364 and PTP4A2 mRNA expression in CC cells subjected to RNase R and actinomycin D (2 μg/ml) treatment. *p < 0.05

To determine circRNAs profiling in CC, we analyzed CC microarray gene profiling datasets GSE113696 and GSE102686 [8]. First, we identified the top 250 differentially expressed (DE) circRNAs using GEO2R, using *P* < 0.05 and fold control (FC) ≥2 as cutoff criteria. Next, we identified 20 DE circRNAs (8 upregulated, 12 downregulated) by combining the above datasets. Of the 8 upregulated circRNAs (Figure 1(a)), we elected to focus on the novel circRNA hsa_circ_0007364 (hsa_circRNA_100141), which is generated from exon2 and exon3 of PTP4A2 gene through back-splicing (Figure 1(b)). Further analysis revealed that hsa_circ_0007364 reverse-transcribed using Oligo dT primers was less than from random primers (Figure 1(c)) [22], hsa_circ_0007364 is more resistant to RNase R digestion relative to linear PTP4A2 mRNA (Figure 1(d)), and hsa_circ_0007364 is markedly more stable than PTP4A2 mRNA following transcription inhibition with actinomycin D (Figure 1(e)).

**Figure 2.**
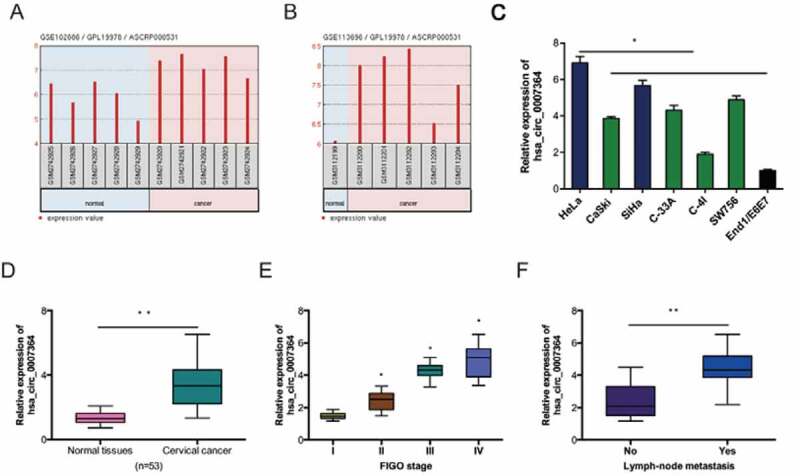
Hsa_circ_0007364 is overexpressed in CC. (a, b) Hsa_circ_0007364 expression in GSE102686 and GSE113696 datasets. (c, d) Hsa_circ_0007364 expression in CC cell lines and tissues. (e, f) High hsa_circ_0007364 expression in CC patients correlates with advanced FIGO stage and lymph-node metastasis. *p < 0.05

Assessment of hsa_circ_0007364 expression in CC revealed that hsa_circ_0007364 is remarkably upregulated in GSE102686 and GSE113696 datasets (Figure 2(a,b)). And the results were further confirmed in CC cell lines and tissues (Figure 2(c,d)). Next, analysis of correlation between hsa_circ_0007364 expression and clinical features showed that high elevated hsa_circ_0007364 levels correlated with advanced FIGO stage and lymph-node metastasis in CC patients (Figure 2(e,f)).

### 3.2. Hsa_circ_0007364 silencing suppresses the invasion and proliferative ability of CC cells

**Figure 3.**
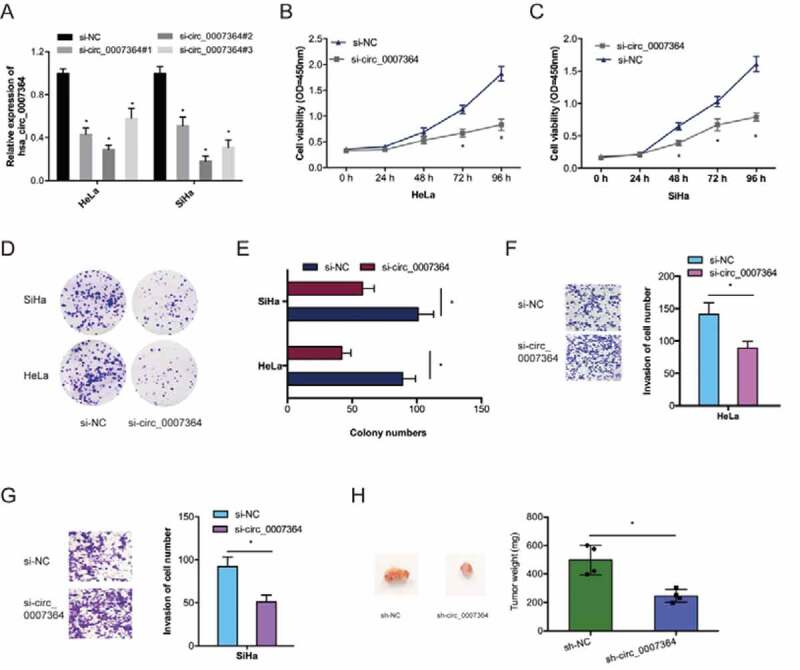
Hsa_circ_0007364 promotes the invasion and proliferative ability of CC cells. (a) Hsa_circ_0007364 knockdown efficiency in CC cells. (b–e) CCK-8 and colony formation assays were used to assess cell proliferation rate *in vitro*. (f, g) Transwell assay was performed to assess cell invasion *in vitro*. (h) hsa_circ_0007364 knockdown suppresses tumor growth *in vivo*. *p < 0.05

To examine the roles of hsa_circ_0007364 on CC progression, we silenced it using si-circ_0007364 in HeLa and SiHa cells (Figure 3(a)). CCK-8 analysis revealed reduced CC cell proliferation upon hsa_circ_0007364 silencing relative to si-NC group (Figure 3(b,c)). Similar observations were made via colony formation analysis (Figure 3(d,e)). Transwell invasion assays showed hsa_circ_0007364 silencing suppressed CC cell invasion *in vitro* (Figure 3(f,g)). Moreover, xenograft experiments revealed that hsa_circ_0007364 knockdown suppresses CC growth *in vivo* (Figure 3(h)). Together, these data indicated that hsa_circ_0007364 silencing could reduce CC progression.

### 3.3. miR-101-5p is a direct target of hsa_circ_0007364

**Figure 4.**
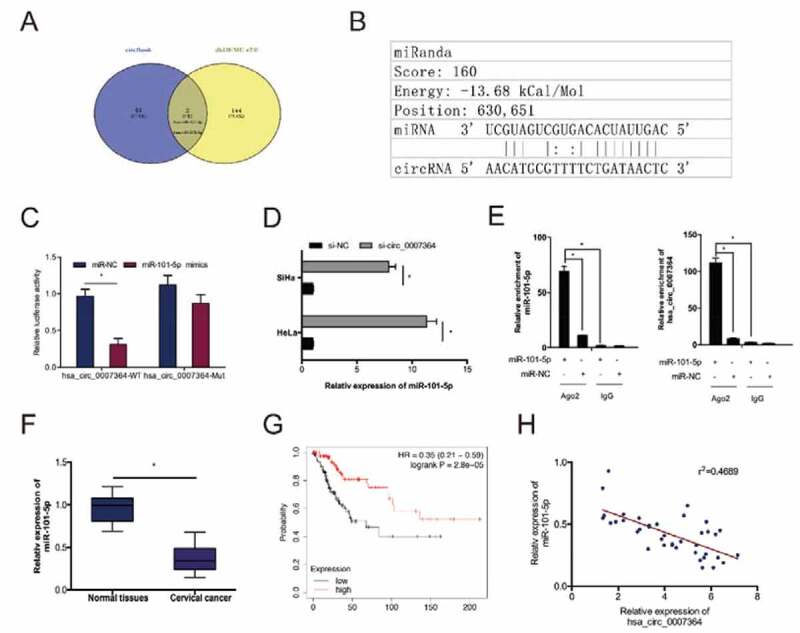
miR-101-5p is a direct target of hsa_circ_0007364. (a) The result of Venn diagram. Overlapping sections represent miRNAs identified by both circBank and dbDEMC v2.0 database. (b) MiR-101-5p has sites with complementarity to hsa_circ_0007364. (c) MiR-101-5p mimics suppress luciferase activity of hsa_circ_0007364-WT group. (d) Hsa_circ_0007364 suppression increases miR-101-5p expression in CC cells. (e) RIP analysis confirmed the interaction between miR-101-5p and hsa_circ_0007364. (f, g) MiR-101-5p downregulation correlates with poor CC patient survival. (h) MiR-101-5p expression negatively correlates with hsa_circ_0007364 levels in CC. *p < 0.05

Previous studies have shown that circRNAs can ‘sponge’ miRNAs in CC cells [23]. To establish whether hsa_circ_0007364 has this function, we predicted its putative target miRNAs using circBank and dbDEMC v2.0. This analysis identified miR-101-5p as a hsa_circ_0007364 target (Figure 4(a,b)). A dual-luciferase reporter analysis indicated that miR-101-5p mimics remarkably decreased luciferase activity in hsa_circ_0007364-WT transfected cells (Figure 4(c)). RT-qPCR analysis revealed hsa_circ_0007364 silencing elevates miR-101-5p expression in HeLa and SiHa cells (Figure 4(d)). The association between hsa_circ_0007364 and miR-101-5p was further confirmed by RIP analysis (Figure 4(e)). RT-qPCR analysis revealed reduced miR-101-5p expression in CC tissues, which correlates with poor overall survival in CC patients (Figure 4(f,g)). Moreover, correlation analysis indicated that low miR-101-5p expression negatively correlated with hsa_circ_0007364 levels in CC tissues (Figure 4(h)). These data suggested that miR-101-5p is a target of hsa_circ_0007364 in CC.

### 3.4. MAT2A is a target of miR-101-5p in CC

**Figure 5.**
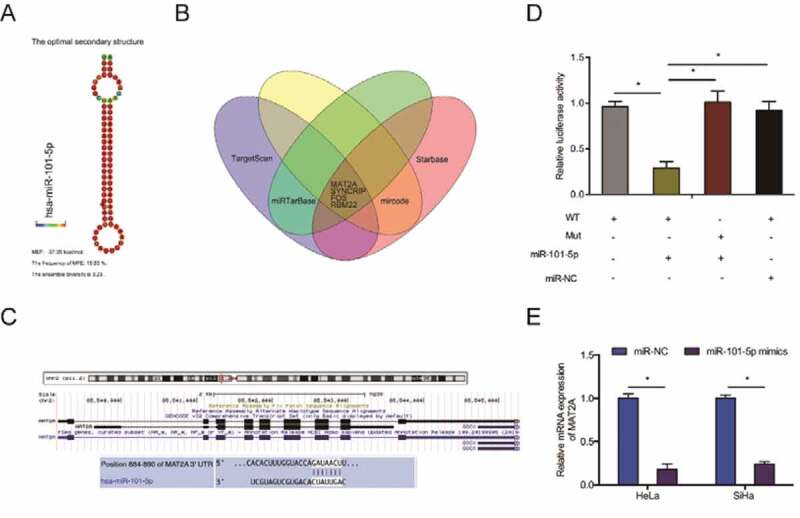
MAT2A is miR-101-5p target in CC. (a) The secondary structure of miR-101-5p. (b) Overlapping miR-101-5p target mRNAs identified TargetScan, Starbase, miRTarBase and mircode. (c) Schematic of the predicted MAT2A binding site on miR-101-5p. (d) MiR-101-5p mimics suppress luciferase activity of MAT2A-WT transfected cells. (e) MiR-101-5p mimics suppress MAT2A expression in CC cells. *p < 0.05

To elucidate the mechanism of miR-101-5p activity in CC cells, we predicted its targets using TargetScan, Starbase, miRTarBase and mircode and identified MAT2A as a possible target for miR-101-5p (Figure 5(a–c)). Dual-luciferase reporter analysis indicated that miR-101-5p mimics decreased the luciferase activity of MAT2A-WT transfected cells (Figure 5(d)). RT-qPCR analysis indicated that miR-101-5p mimics suppress MAT2A expression in CC cells (Figure 5(e)).

**Figure 6.**
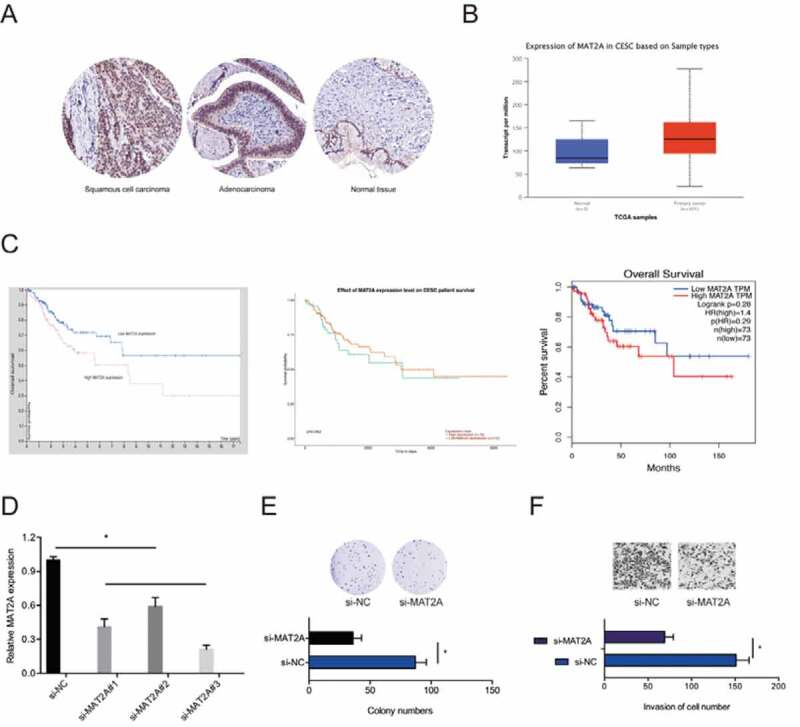
The roles of MAT2A in CC. (a, b) MAT2A expression in CC tissues was examined by IHC and TCGA datasets. (c) High MAT2A expression was related to poor disease outcomes in CC patients. (d) MAT2A knockdown efficiency in HeLa cells. (e, f) MAT2A inhibition decreased HeLa cells proliferation and invasion ability *in vitro*. *p < 0.05

Next, we determined MAT2A expression in CC. IHC revealed that MAT2A expression is markedly upregulated in CC (Figure 6(a)). Similar results were obtained from analysis of TCGA datasets (Figure 6(b)). Kaplan-Meier analysis indicated that high MAT2A expression was significantly associated with poor CC prognosis (Figure 6(c)). Next, we transfected si-MAT2A into HeLa cells (Figure 6(d)). Colony formation and transwell invasion assays revealed that MAT2A inhibition significantly decreased the proliferative and invasion ability of HeLa cells *in vitro* (Figure 6(e,f)). Taken together, these data showed that MAT2A is a downstream target of miR-101-5p in CC.

### 3.5. Hsa_circ_0007364 promotes CC progression via the miR-101-5p/MAT2A axis

**Figure 7.**
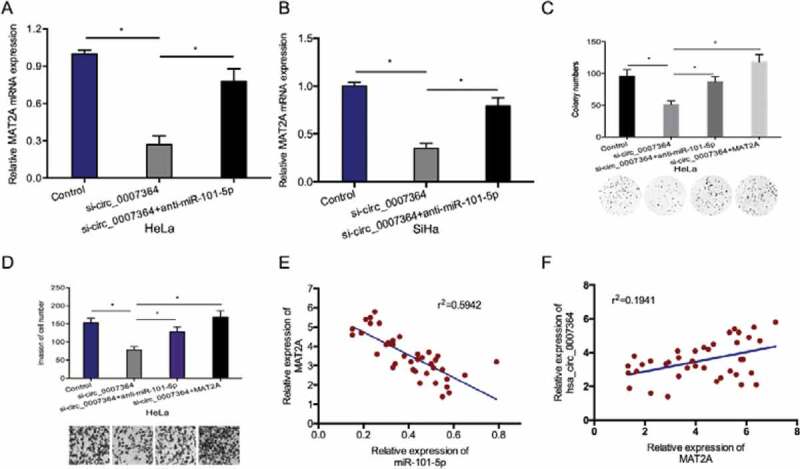
Hsa_circ_0007364 promotes CC progression via the miR-101-5p/MAT2A axis. (a, b) MiR-101-5p suppression blocked the effects of hsa_circ_0007364 silencing on MAT2A expression. (c, d) MAT2A overexpression (or miR-101-5p suppression) abolished the effects of hsa_circ_0007364 silencing on HeLa cells colony formation and invasion *in vitro*. (e) MAT2A expression negatively correlates with miR-101-5p expression in CC tissues. (f) MAT2A expression positively correlates with hsa_circ_0007364 expression in CC tissues. *p < 0.05

Next, we examined whether hsa_circ_0007364 functions as a molecular sponge against miR-101-5p to regulate MAT2A expression. RT-qPCR analysis revealed that hsa_circ_0007364 deficiency resulted in reduced MAT2A expression, while miR-101-5p suppression reversed this effect (Figure 7(a,b)). Rescue assays showed that MAT2A overexpression (or miR-101-5p suppression) abolishes the effects of silencing hsa_circ_0007364 on HeLa cell proliferation and invasion (Figure 7(c,d)). Moreover, correlation analysis indicated that elevated MAT2A expression inversely correlates with miR-101-5p expression and positively correlates with hsa_circ_0007364 expression in CC tissues (Figure 7(e–f)). Taken together, these data showed that hsa_circ_0007364 might regulate MAT2A expression by sponging miR-101-5p in CC.

## 4. Discussion

Cervical cancer (CC) is one of the commonest malignancies globally [2]. Mounting evidence shows that aberrant expression of circRNA is closely associated with cancer development, including CC [24]. However, the functions of circRNAs in CC remain poorly studied. Here, we identified a novel circRNA hsa_circ_0007364, derived from RNA cyclization following a head-to-tail splicing of exon 2 and exon 3 of PTP4A2 (protein tyrosine phosphatase 4A2). PTP4A2 has been reported to influence tumor progression and stem cell self-renewal [25–27]. Here, we found that hsa_circ_0007364 is remarkably overexpressed and correlates with advanced FIGO stage and lymph-node metastasis in CC patients. At a functional level, our data showed that hsa_circ_0007364 facilitates CC proliferation and invasion, suggesting that it may have oncogenic functions in CC.

Multiple studies show that circRNAs participate in tumorigenesis by sponging miRNAs [28,29]. Prediction of hsa_circ_0007364 putative targets using circBank uncovered miR-101-5p as possible target. Interaction between hsa_circ_0007364 and miR-101-5p was confirmed using a dual-luciferase reporter and RIP assay. MiR-101-5p delays tumor growth in various cancers, including liver cancer, lung cancer and clear cell renal cell carcinoma [11–13]. In agreement with these reports, we showed that miR-101-5p expression is suppressed in CC tissues, and that miR-101-5p suppression abated the suppression of the proliferative and invasion ability of CC cells caused by hsa_circ_0007364 silencing. These data suggested that hsa_circ_0007364 might exert its functions by sponging miR-101-5p in CC.

MiRNAs modulate various cellular processes via their molecular targets [30]. A search for possible miR-101-5p targets using TargetScan, Starbase, miRTarBase and mircode software uncovered MAT2A as a direct miR-101-5p target. MAT2A is implicated in various human cancers, including hepatocellular carcinoma, and gastric cancer [15,16,31]. However, the roles of MAT2A in CC have not been investigated. Here, we found that MAT2A is overexpressed in CC and related to poor disease outcome. Furthermore, MAT2A upregulation reversed the suppression of proliferation and invasion caused by hsa_circ_0007364 silencing in HeLa cells. Moreover, we show that MAT2A is positively modulated by hsa_circ_0007364 and negatively modulated by miR-101-5p. Together, these data suggest that hsa_circ_0007364 exerts its oncogenic activity via the miR-101-5p/MAT2A axis.

## 5. Conclusions

In summary, we found that hsa_circ_0007364 is overexpressed in CC, and may enhance the proliferative and invasion capacity of CC cells by interacting with the miR-101-5p/MAT2A axis. Our findings highlight this axis as a potential therapeutic target against CC.

## Data Availability

The datasets supporting the conclusions of this article are included within the article.
